# CD147-high classical monocytes: a cellular biomarker for COVID-19 disease severity and treatment response

**DOI:** 10.1186/s41232-025-00371-8

**Published:** 2025-04-07

**Authors:** Teruaki Murakami, Yuta Yamaguchi, Saori Amiya, Yuko Yoshimine, Shinichiro Nameki, Yasutaka Okita, Yasuhiro Kato, Haruhiko Hirata, Yoshito Takeda, Atsushi Kumanogoh, Takayoshi Morita

**Affiliations:** 1 Department of Respiratory Medicine and Clinical Immunology, Graduate School of Medicine, Osaka University, 2-2, Yamadaoka, Suita, Osaka 565-0871 Japan; 2 Department of Immunopathology, Immunology Frontier Research Center (WPI-IFReC), Osaka University, Suita, 565-0871 Japan; 3 Division of Pharmacology, Graduate School of Medicine, Kobe University, Kobe, Hyogo Japan; 4 Medical Center for Translational Research, Department of Medical Innovation, Osaka University Hospital, Osaka, Japan; 5 Integrated Frontier Research for Medical Science Division, Institute for Open and Transdisciplinary Research Initiatives (OTRI), Osaka University, Suita, 565-0871 Japan; 6 Center for Infectious Disease Education and Research (CiDER), Osaka University, Suita, 565-0871 Japan; 7 Japan Agency for Medical Research and Development – Core Research for Evolutional Science and Technology (AMED–CREST), Osaka University, Suita, Osaka Japan; 8 Center for Advanced Modalities and DDS, Osaka University, Suita, Osaka Japan; 9 Strategic Global Partnership & X(Cross)-Innovation Initiative, Graduate School of Medicine, Osaka University and Osaka University Hospital, Osaka, Japan

**Keywords:** COVID-19, SARS-CoV-2, Spike S1 subunit, CD147, Classical monocyte, Cytokine storm, Inflammation, Mass cytometry

## Abstract

**Background:**

Severe acute respiratory syndrome coronavirus 2 (SARS-CoV-2) infection can lead to severe coronavirus disease 2019 (COVID-19), which is characterized by cytokine storm and organ dysfunction. The spike S1 subunit induces inflammatory cytokine production, but the immune cell subsets that respond to S1 stimulation and contribute to disease severity remain unclear.

**Methods:**

We analyzed serum samples and peripheral blood mononuclear cells (PBMCs) from patients with COVID-19 (moderate: *n* = 7; severe: *n* = 25) and healthy controls (*n* = 38). Using mass cytometry (cytometry by time-of-flight; CyTOF), we analyzed immune cell responses to S1 subunit stimulation in PBMCs from healthy donors and patients with COVID-19. We examined correlations among identified cell populations, serum cytokine levels, and clinical parameters.

**Results:**

Serum S1 subunit levels correlated with disease severity and inflammatory cytokine concentrations. S1 subunit stimulation induced dose-dependent cytokine production from PBMCs, predominantly from myeloid cells. CyTOF analysis identified classical monocytes with high CD147 expression (CD147hi cMono) as the primary source of S1-induced cytokines. The proportion of CD147hi cMono increased significantly in severe COVID-19 and decreased with clinical improvement. The frequency of CD147hi cMono showed a stronger positive correlation with clinical severity markers in younger patients compared to older patients.

**Conclusions:**

CD147hi cMono are the primary cellular source of S1-induced inflammatory cytokines and may serve as potential biomarkers for monitoring COVID-19 severity and treatment response.

**Supplementary Information:**

The online version contains supplementary material available at 10.1186/s41232-025-00371-8.

## Background

The severe acute respiratory syndrome coronavirus 2 (SARS-CoV-2) pandemic emerged as a major global health challenge, with over 777 million confirmed cases and more than 7 million deaths worldwide as of November 2024 [1, 2]. Severe Coronavirus disease 2019 (COVID-19) is characterized by a hyperinflammatory response, known as a cytokine storm, which contributes to the development of acute respiratory distress syndrome (ARDS), multi-organ dysfunction, and increased mortality [3, 4].

The SARS-CoV-2 spike S1 subunit plays crucial roles in viral pathogenesis [5–7]. While it primarily mediates viral entry through binding to angiotensin-converting enzyme 2 [5], the S1 subunit also triggers inflammatory responses through toll-like receptors (TLRs), including TLR2, TLR4, and TLR6 [6, 7]. These receptors, highly expressed on monocytes and macrophages, initiate inflammatory cytokine production upon stimulation by the S1 subunit. However, the subsets of monocytes and other immune cells that are responsible for S1 subunit-induced cytokine production in severe COVID-19 remain undefined.

CD147, also known as basigin or extracellular matrix metalloproteinase inducer (EMMPRIN), mediates inflammatory cytokine production and plays multiple roles in inflammatory responses [8, 9]. In COVID-19 research, CD147 has mainly been investigated as a potential receptor for SARS-CoV-2, particularly through its interaction with S1 subunit [10–12], but its role in inflammation during COVID-19 remains poorly understood. In support of the clinical importance of CD147, recent clinical trials using a humanized anti-CD147 antibody have shown promising results in patients with severe COVID-19, demonstrating reduced mortality, viral load, and cytokine levels [13, 14].

This study aimed to identify immune cell populations responding to S1 subunit stimulation and examine their association with COVID-19 severity. We focused particularly on CD147-expressing cells, given their potential role in inflammatory responses and as therapeutic targets. Using mass cytometry analysis of peripheral blood mononuclear cells (PBMCs), we investigated the cellular sources of inflammatory cytokines and their correlation with clinical parameters in patients with COVID-19.

## Materials and methods

### Study design

We conducted a prospective observational study at Osaka University Hospital from July 2020 to February 2021. The study included 32 patients with PCR-confirmed COVID-19 admitted to the intensive care unit (ICU) and 38 healthy donors (HDs) serving as controls. COVID-19 patients (median age 73 years [IQR: 61.75–79]; 75% male) and HDs (median age 48 years [IQR: 41–53.5]; 43.59% male) were enrolled following institutional protocols. Disease severity was classified according to the WHO Ordinal Scale for Clinical Improvement [15]: moderate (oxygen by mask or nasal prongs, *n* = 7) and severe (intubation and mechanical ventilation, *n* = 25). For 17 patients, we collected additional samples at ICU discharge. All participants were unvaccinated and provided written informed consent. This study was approved by the Ethics Committee of Osaka University Graduate School of Medicine (No. 18050).

### Patient enrollment and sample collection

Blood samples were collected in heparin-coated tubes for PBMC isolation and in plain tubes for serum collection. PBMCs were isolated by density gradient centrifugation using Leucosep (Greiner, Germany). Isolated PBMCs were cryopreserved in CELLBANKER medium (Nippon Zenyaku Kogyo Co., Japan) and stored in liquid nitrogen. For serum preparation, blood samples were allowed to clot for 15 min at room temperature (RT) and centrifuged at 1000 g for 15 min. Serum samples were stored at − 80°C until analysis. Clinical characteristics and laboratory data were collected from electronic medical records (Table S1).

### Measurement of serum S1 subunit levels

We measured serum SARS-CoV-2 spike S1 subunit concentrations using the SARS-CoV-2 Spike Protein Titer Assay Kit (Acrobiosystems, China). Briefly, serum samples or standards (100 μL) were incubated in anti-SARS-CoV-2 spike antibody-coated plates for 1 h at 37℃. After washing, horseradish peroxidase (HRP)-conjugated anti-SARS-CoV-2 spike antibody was added and incubated for 1 h at 37℃. Following substrate reaction (20 min, 37℃), absorbance was measured at 450 nm (reference: 630 nm) using a SpectraMax i3x reader (Molecular Devices, USA). Sample concentrations were calculated using the standard curve.

### Ex vivo PBMC stimulation with S1 subunit

Cryopreserved PBMCs from HDs were thawed, washed with Roswell Park Memorial Institute (RPMI) (Nacalai Tesque, Japan) containing 10% heat-inactivated fetal bovine serum (FBS) (Thermo Fisher Scientific, USA), and cultured (1 × 10^5^ cells/well) with SARS-CoV-2 spike S1 subunit (Acrobiosystems, China) at 0, 1.25, 2.5, or 5 μg/mL for 24 h. Culture supernatants were collected and stored at −80℃ for cytokine measurement.

### Measurement of cytokine production

Cytokine levels in culture supernatants were measured using LEGENDplex assay (Biolegend, USA). Mixed capture beads and assay buffer were incubated with samples or standards in a 96-well V-bottom plate (2 h, RT, dark). After washing, detection antibodies were added (1 h, RT), followed by streptavidin–phycoerythrin (30 min, RT). Data was acquired using FACSCanto II (BD Biosciences) and analyzed using LEGENDplex software. Measurements below detection limits were assigned the minimum detectable value.

### Mass cytometry analysis

#### Experimental Procedures

##### Mass cytometry antibodies

We purchased most antibodies from Standard Biotools. Some antibodies from R&D Systems were metal-tagged in our laboratory using the MaxPar X8 polymer kit (Standard Biotools, USA) with Praseodymium-141, Neodymium-144, Terbium-159, Gadolinium-160, Thulium-169, or Ytterbium-176. For cytokine analysis in S1 subunit stimulation experiments, we employed an extended panel of 40 antibodies against both cell surface markers and intracellular cytokines (Table S2). For cell surface marker analysis of patient samples, we used a panel of 27 metal-tagged antibodies (Table S3). All antibodies were stored in stabilizer solution (Candor Bioscience, Germany) at 4℃.

##### Staining procedures

For intracellular cytokine analysis of S1 subunit-stimulated samples, PBMCs were barcoded using anti-CD45 antibodies conjugated to different metal isotopes (89Y, 106Cd, 110Cd, 111Cd, 113Cd, 114Cd, and 116Cd) for sample multiplexing. After barcode staining (30 min, RT) and washing, cells were labeled with Cell-ID Cisplatin-198Pt (5 min, RT). Following FcR blocking (10 min, 4°C), cells were stained with metal-conjugated surface antibodies (30 min, RT), then fixed and permeabilized using Foxp3 Transcription Factor Staining Buffer Set (Thermo Fisher Scientific), followed by staining with cytokine antibodies according to Table S2.

For cell surface marker analysis of COVID-19 patient samples, PBMCs were barcoded using anti-CD45 antibodies conjugated to different metal isotopes (89Y, 106Cd, 110Cd, 111Cd, 112Cd, 113Cd, 114Cd, and 116Cd) for sample multiplexing. After barcode staining (30 min, RT) and washing, cells were labeled with Cell-ID Cisplatin-198Pt (5 min, RT). Following FcR blocking (10 min, 4 °C), cells were stained with metal-conjugated cell surface antibodies (30 min, RT).

##### Data acquisition

Stained cells were washed with Cell Staining Buffer and Cell Acquisition Solution (CAS). Samples were adjusted to 2 × 10^6^ cells/mL in CAS containing 15% EQ Four Element Calibration Beads, filtered (35 μm), and analyzed using a Helios mass cytometer (200–300 cells/sec). Data files were normalized using the Standard Biotools normalizer software.

### Data analysis

#### Analysis of S1 subunit stimulation experiments

Live single cells were gated using Cytobank. Data analysis was performed in R (v4.2.3) using CATALYST (v1.14.0) for normalization and de-barcoding, and cytofworkflow (v1.24.0) for clustering, dimensional reduction, and visualization [16]. The clustering of all PBMCs was conducted using markers listed in Table S2. For monocyte re-clustering, the markers CD16, CD38, CD14, CD11c, CD45RA, HLA-DR, and CD147 were used.

#### Analysis of COVID-19 patient samples

As patient samples were acquired in multiple batches, we first performed batch alignment using cyCombine (v0.2.15) [17]. After alignment, live single cells were gated using Cytobank, followed by analysis in R using CATALYST for normalization and de-barcoding, and CyTOF workflow packages for clustering, dimensional reduction, and visualization. PBMC clustering utilized markers including CD16, CD27, CD161, CD38, CD45RA, CXCR5, CD14, and others described in Table S3. For monocyte sub-clustering, CD16, CD38, CD14, CD11c, CD45RA, HLA-DR, and CD147 were utilized. Visualizations were created using ggplot2 (v3.3.5).

### Statistical analysis

Analyses were performed using rstatix (v0.7.0) or GraphPad Prism (v10.2.1). Student's t-test or the Mann–Whitney U test was used for two-group comparisons on the basis of data distribution. For multiple group comparisons, one-way ANOVA with Tukey's post-hoc test or the Kruskal–Wallis test with Dunn's test was used. Correlations were assessed by Spearman's rank correlation. To address the potential confounding effect of age, a stratified analysis was performed. Previous studies have shown that adults over 65 years of age represent 80% of COVID-19 hospitalizations and have a greater risk of death compared to those under 65 [18]. Therefore, to account for this potential confounding effect, patients were divided into two age groups: < 65 years and ≧65 years. Spearman's rank correlation coefficients were calculated between CD147hi cMono frequency and other variables of interest separately within each age group. Statistical significance was defined as p < 0.05 (* , *p* < 0.05; ∗ ∗ , *p* < 0.01; ∗ ∗ ∗ , *p* < 0.001).

## Results

### Serum S1 subunit and cytokine levels correlate with COVID-19 severity

To investigate the relationship between the SARS-CoV-2 spike S1 subunit and COVID-19 severity, we enrolled 32 patients with COVID-19 admitted to the intensive care unit (ICU) of Osaka University Hospital (median age 73 years [IQR; 61.75–79]; male, 75.0%) and 38 healthy donors (HDs) as controls (median age 48 years [IQR; 41–53.5]; male, 43.59%). Among COVID-19 patients, 7 had moderate disease requiring oxygen by mask or nasal prongs, and 25 had severe disease requiring intubation and mechanical ventilation based on WHO Ordinal Scale for Clinical Improvement. We collected peripheral blood samples, demographic data, and clinical parameters, including duration of mechanical ventilation and corticosteroid treatment, from electronic medical records at ICU admission (Table S1).

**Fig. 1:**
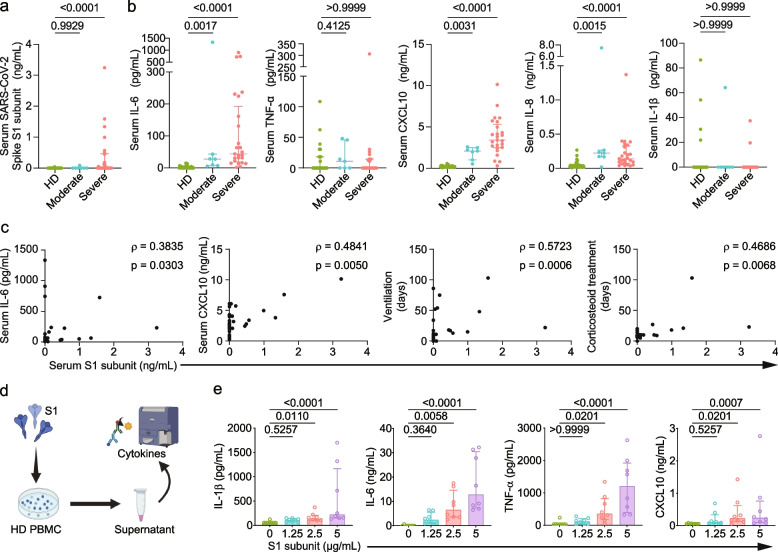
S1 subunit levels and cytokine production correlate with COVID-19 severity. **a**. Serum levels of S1 subunit in patients with COVID-19 (moderate: *n* = 7; severe: *n* = 25) and healthy donors (HDs, *n* = 38). Severity was classified by the WHO Ordinal Scale for Clinical Improvement. Significance was determined by the Kruskal–Wallis test, and bars indicate medians. **b**. Levels of cytokines in patients with COVID-19 and HDs. Each value is indicated by a dot and the median by a bar. Pairwise comparisons were performed using the Kruskal–Wallis test. **c**. Correlation analysis between serum S1 subunit concentration and clinical parameters, hematological data, and serum cytokines. Each graph shows Spearman's correlation coefficient and *p*-value. **d**. Schematic overview of PBMC stimulation with S1 subunit. **e**. Cytokine levels in culture supernatants following S1 stimulation. Significance was determined by the Kruskal–Wallis test, and the bars indicate medians

Serum S1 subunit levels were significantly elevated in patients with severe COVID-19 compared with those with moderate disease and HDs (Fig. 1a). Serum cytokine analysis revealed significantly higher levels of interleukin (IL)−6, tumor necrosis factor (TNF)-α, C-X-C motif chemokine ligand (CXCL)10, and IL-8 in patients with COVID-19, particularly in severe cases, compared to HDs. Although serum IL-1β levels were also measured, no significant difference was observed between the groups (Fig. 1b and Supplementary Fig. 1a). Furthermore, we evaluated correlations between these cytokines, clinical parameters, and S1 subunit concentration. The serum S1 subunit level showed significant positive correlations with serum IL-6 (*ρ* = 0.3835, *p* = 0.0303), CXCL10 (*ρ* = 0.4841, *p* = 0.0050), duration of mechanical ventilation (*ρ* = 0.5723, *p* = 0.0006), and corticosteroid treatment period (*ρ* = 0.4686, *p* = 0.0068) (Fig. 1c and Supplementary Fig. 1b). Additional correlations between S1 subunit levels and other clinical parameters are shown in Supplementary Fig. 1b. These findings demonstrate that serum S1 subunit levels are associated with both inflammatory markers and clinical severity of COVID-19.

### Myeloid cells predominantly produce inflammatory cytokines in response to S1 subunit

**Fig. 2:**
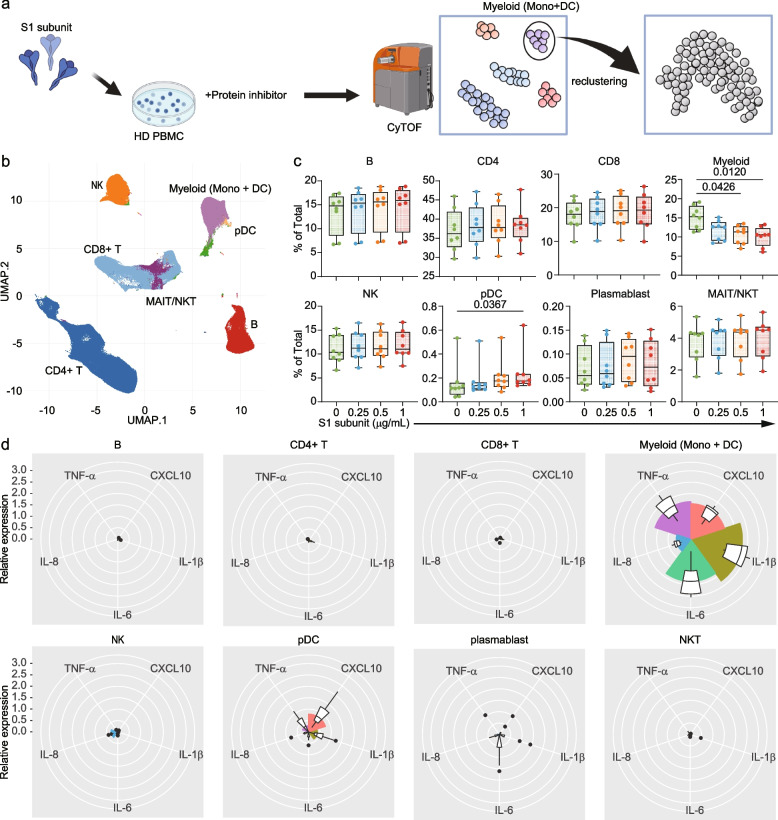
Myeloid cells respond to S1 subunit and produce inflammatory cytokines. **a**. Experimental workflow for intracellular cytokine analysis using mass cytometry. **b**. UMAP visualization of major immune populations. **c**. Changes in immune subset proportions following S1 subunit stimulation. Significance was determined by the Kruskal–Wallis test. **d**. Analysis of cytokine expression (CXCL10, IL-1β, IL-6, IL-8, and TNF-α) in major immune populations following stimulation with 0 or 1 μg/mL of S1 subunit. The color intensity represents the log2-fold change in expression

To explore the role of S1 subunit in hyperinflammation observed in COVID-19, we stimulated PBMCs from 8 healthy donors with various concentrations of recombinant S1 subunit (Fig. 1d). We observed dose-dependent increases in IL-1β, IL-6, TNF-α, and CXCL10 levels (Fig. 1e and Supplementary Fig. 1c). To determine which immune cell subsets in PBMCs produce cytokines in response to S1 subunit stimulation, we performed cytometry by time of flight (CyTOF) analysis (Fig. 2a). We identified 8 major immune subsets of CD45 positive viable cells using FlowSOM (Self-Organizing Map clustering and Minimal Spanning Trees) (Fig. 2b and Supplementary Fig. 2a). After S1 subunit stimulation, the proportion of myeloid cells and plasmacytoid dendritic cells were significantly decreased and increased in accordance with the S1 subunit concentrations, respectively (Fig. 2c). We identified myeloid cells as the main source of proinflammatory cytokines in PBMCs in response to S1 subunit stimulation (Fig. 2d).

### CD147hi classical monocytes are the primary source of S1 subunit-induced cytokines

**Fig. 3:**
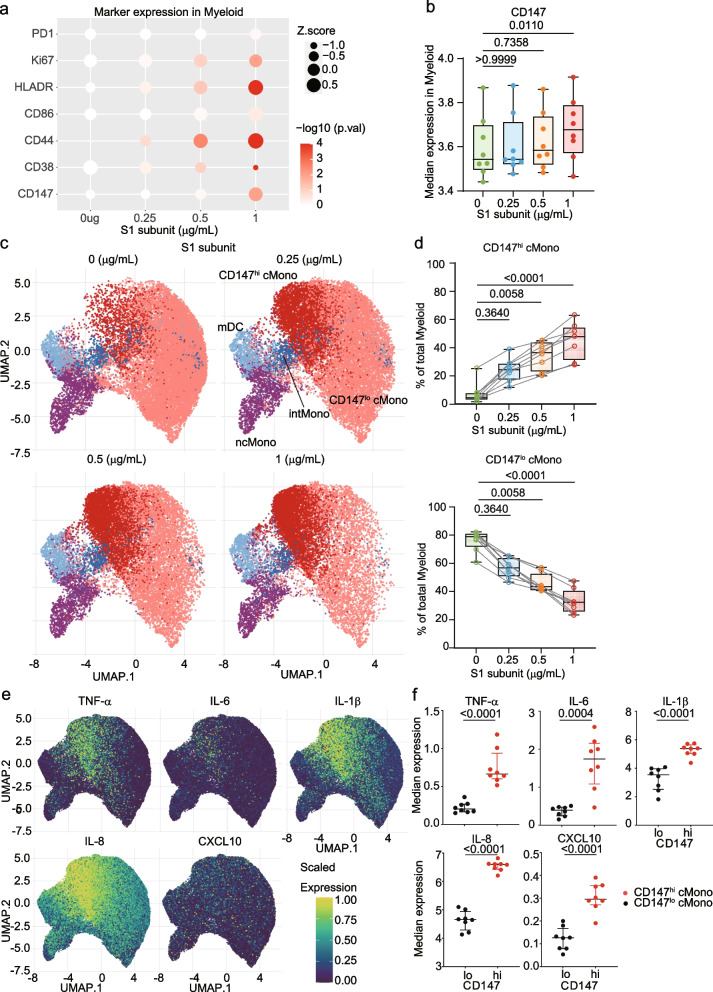
CD147hi classical monocytes produce cytokines in response to S1 subunit. **a**. Expression of activation markers and CD147 in myeloid cells. The bubble size represents the Z score and color intensity indicates the -log10 *p*-value. **b**. CD147 expression levels on myeloid cells. Box plots show interquartile ranges. Significance was determined by the Kruskal–Wallis test. **c**. UMAP visualization of myeloid cells showing CD147 expression following S1 stimulation (0, 0.25, 0.5, and 1 μg/mL). **d**. Proportion of CD147hi and CD147lo cMono in response to S1 subunit stimulation. Significance was determined by the Kruskal–Wallis test. **e**. Expression of TNF-α, IL-6, IL-1β, IL-8, and CXCL10 in myeloid populations shown by UMAP. **f**. Cytokine expression levels in CD147hi and CD147lo cMono. Significance was determined by the Kruskal–Wallis test, and bars indicate medians

To characterize the myeloid cell subsets responding to S1 subunit, we first analyzed the expression of activation and proliferation markers in CyTOF data. Bubble plot analysis revealed that expression levels of multiple markers, including CD86, HLA-DR, CD44, CD38, and Ki-67, were upregulated in myeloid cells in a concentration-dependent manner following S1 stimulation (Fig. 3a). Notably, CD147 expression also increased significantly with S1 stimulation, showing similar kinetics to the activation markers (Fig. 3a and b, *p* < 0.001 at 1 μg/mL compared to baseline, Supplementary Fig. 2b).

Further analysis of CD147 expression patterns across myeloid subsets showed that classical monocytes (cMono) and intermediate monocytes (intMono) expressed significantly higher levels of CD147 compared to non-classical monocytes (ncMono) and myeloid dendritic cells (mDC) (Supplementary Fig. 2c). Re-clustering analysis of myeloid cells using FlowSOM revealed distinct populations of CD147-high and CD147-low classical monocytes (CD147hi and CD147lo cMono) (Supplementary Fig. 2d). Upon S1 stimulation, the proportion of CD147hi cMono increased significantly (*p* < 0.0001 at 1 μg/mL), while CD147lo cMono showed corresponding decrease (Fig. 3c and d). The proportions of other populations (intMo, ncMo, and mDC) are shown in Supplementary Fig. 2e.

To determine whether these CD147-defined subsets differ in function, we performed intracellular cytokine staining. CD147hi cMono exhibited markedly higher expression of multiple inflammatory cytokines compared to CD147lo cMono, including TNF-α (*p* < 0.0001), IL-6 (*p* < 0.0001), IL-1β (*p* < 0.0001), IL-8 (*p* < 0.0001), and CXCL10 (*p* < 0.0001) (Fig. 3e and f). These findings identify CD147hi cMono as the primary cellular source of S1 subunit-induced inflammatory cytokines.

### CD147hi classical monocytes associated with COVID-19 severity

**Fig. 4:**
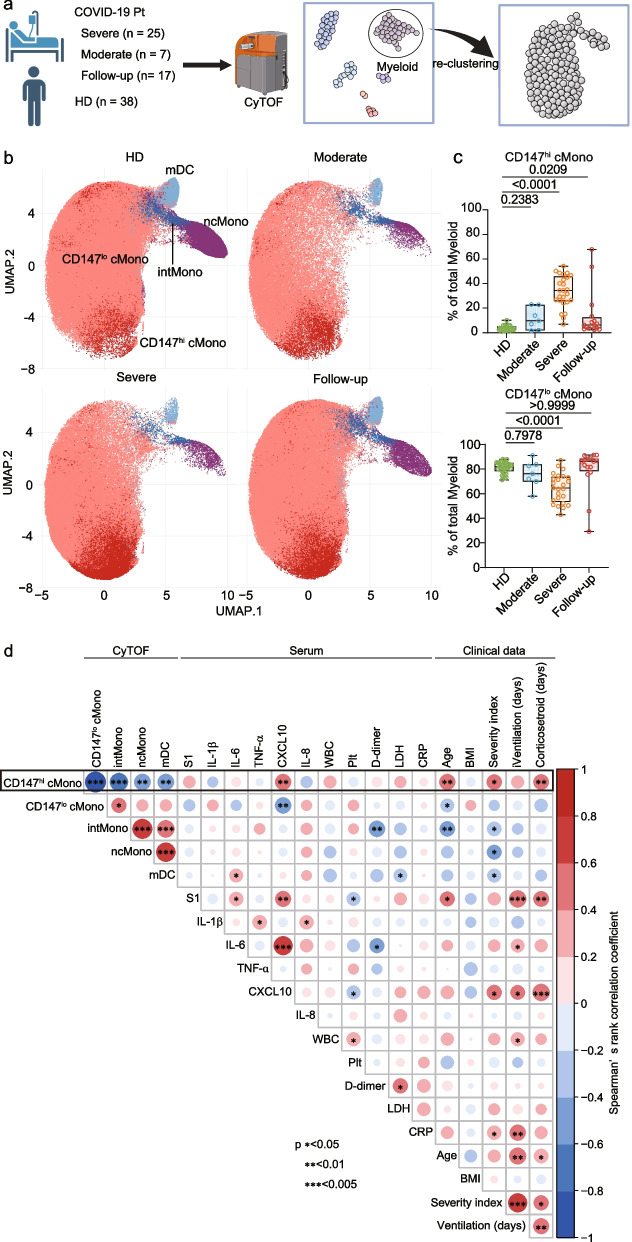
CD147hi classical monocytes correlate with COVID-19 severity. **a**. Overview of mass cytometry analysis of patient samples. Follow-up samples were collected at ICU discharge. **b**. UMAP visualization of myeloid populations highlighting CD147 expression. **c**. Frequency of CD147hi and CD147lo cMono across patient groups. Significance was determined by the Kruskal–Wallis test. **d**. Correlation analysis between myeloid cell frequencies, serum cytokines, and clinical parameters. Heatmap shows Spearman's correlation coefficients, and asterisks indicate *p*-values (*p < 0.05, **p < 0.01, ***p < 0.001)

To examine whether CD147hi cMono are clinically relevant in COVID-19, we analyzed PBMCs from healthy donors (*n* = 38), patients with moderate (*n* = 7) or severe COVID-19 (*n* = 25) at admission, and follow-up samples at discharge (*n* = 17) using mass cytometry (Fig. 4a). FlowSOM clustering identified eight major immune cell populations (Supplementary Fig. 3a and b). Further analysis of myeloid cells using the markers CD16, CD38, CD14, CD11c, CD45RA, HLA-DR, and CD147 identified four populations (cMono, intMono, ncMono, and mDC) and revealed distinct CD147hi and CD147lo cMono populations (Fig. 4b).

The frequencies of the cell population were analyzed in healthy donors, patients with moderate or severe COVID-19, and patients at follow-up (Fig. 4c and supplementary Fig. 3c). Notably, the frequency of CD147hi cMono was significantly greater in patients with severe COVID-19 than in moderate patients and healthy donors (*p* < 0.0001), while CD147lo cMono showed an inverse pattern (*p* < 0.0001) (Fig. 4c). Notably, in follow-up samples collected at discharge, the proportion of CD147hi cMono decreased significantly compared to admission samples (*p* = 0.0209), suggesting normalization with clinical improvement.

Correlation analysis revealed significant associations between CD147hi cMono frequency and multiple clinical parameters. Strong positive correlations were observed with serum CXCL10 levels (*ρ* = 0.5015, *p* = 0.003457), age (*ρ* = 0.4985, *p* = 0.003687), WHO severity scores (*ρ* = 0.4480, *p* = 0.01014), and duration of corticosteroid treatment (*ρ* = 0.4634, *p* = 0.007559) (Fig. 4d and Supplementary Fig. 3d). We also observed positive correlation trends with serum S1 subunit levels (*ρ* = 0.2881, *p* = 0.1098), white blood cell count (*ρ* = 0.3046, *p* = 0.09004), serum lactate dehydrogenase levels (*ρ* = 0.2658, *p* = 0.1414), and duration of mechanical ventilation (*ρ* = 0.3114, *p* = 0.08279) (Fig. 4d).

### Age-stratified analysis of correlations between CD147hi cMono frequency and clinical variables

To further investigate the influence of age on the correlations between CD147hi cMono frequency and clinical variables, we performed a stratified analysis by age, dividing the patients into two groups: younger patients (<65 years, *n* = 11) and older patients (≧65 years, *n* = 20). Spearman’s rank correlation coefficients between CD147hi cMono frequency and all other variables included in the initial correlation analysis were calculated separately within each age group (Table S4).

In the younger group (< 65 years), we observed significant positive correlations between CD147hi cMono frequency and white blood cells (WBC) (*ρ* = 0.682, *p* = 0.025), lactate dehydrogenase (LDH) (*ρ* = 0.679, *p* = 0.022), and Severity (*ρ* = 0.635, *p* = 0.036). In the older group (≥ 65 years), the correlation between CD147hi cMono frequency and steroid treatment duration was statistically significant (*ρ* = 0.433, *p* = 0.050), and a positive correlation trend was found for CXCL10 (*ρ* = 0.405, *p* = 0.070). These correlations with multiple severity markers suggest that CD147hi cMono could be a potential cellular biomarker for monitoring disease severity and treatment response in COVID-19.

## Discussion

This study reveals three key findings regarding the pathogenesis of severe COVID-19. First, serum S1 subunit levels correlate with disease severity and inflammatory markers. Second, we identified CD147hi cMono as the primary cellular source of inflammatory cytokines in response to S1 subunit stimulation. Third, the proportion of CD147hi cMono significantly increases in severe COVID-19 and correlates with clinical severity markers, suggesting its potential as a disease biomarker. Notably, our stratified analysis further revealed age-specific patterns in the associations between CD147hi cMono frequency and clinical variables, highlighting the complex interplay of age and immune responses in COVID-19 pathogenesis.

CD147 plays multiple roles in inflammatory responses through its associations with signaling molecules including nuclear factor-kappa B (NF-κB), mitogen-activated protein kinase (MAPK), and phosphatidylinositol-3 kinase (PI3K)/ protein kinase B (Akt) [8, 9]. Previous studies have shown that CD147 contributes to cytokine production during viral infections, including human immunodeficiency virus (HIV) and SARS-CoV [19, 20]. Our findings extend these observations by demonstrating that CD147 expression levels on classical monocytes define a subset particularly responsive to SARS-CoV-2 S1 subunit stimulation. In the overall cohort, and more distinctly in the younger age group, the strong correlation between CD147hi cMono frequency and multiple clinical severity markers suggests that these cells may be central to COVID-19 pathogenesis, especially in younger individuals. Given their cytokine profile, CD147hi cMono likely contribute to the lung inflammation seen in severe COVID-19 [3, 4]. CD147 has also been implicated in pulmonary fibrosis [21]. While we focused on circulating monocytes, CD147 is expressed on lung-resident cells such as alveolar macrophages and epithelial cells [22], suggesting potential interactions. Further investigations are needed to elucidate the precise mechanism of S1-mediated activation of CD147hi cMono.

While the precise mechanism of S1-mediated activation of CD147hi cMono requires further investigation, several possibilities exist. It likely involves a combination of direct interaction with CD147 [10] and indirect activation through TLRs, particularly TLR2 and TLR4, which are known to be stimulated by the S1 subunit [6, 7]. These interactions trigger downstream signaling pathways, including NF-κB, MAPK, and PI3K/Akt, leading to inflammatory cytokine production. Furthermore, other studies have shown that the SARS-CoV-2 S protein can activate the NLR family pyrin domain containing 3 (NLRP3) inflammasome in immune cells, providing an additional mechanism for IL-1β and IL-18 production [23]. Future studies, potentially employing CD147 blocking antibodies or genetic knockout approaches, are needed to definitively elucidate the specific contribution of CD147 to S1-mediated cytokine production.

Age is a well-established risk factor for severe COVID-19 [3, 4], and our study also revealed a positive correlation between age and CD147hi cMono frequency. Our stratified analysis revealed distinct age-specific correlation patterns. In the younger group (< 65 years), the CD147hi cMono frequency was significantly positively correlated with the WBC count, LDH level, and disease severity score. These correlations suggest that in younger patients, CD147hi cMono may contribute to systemic inflammation and disease severity. Elevated WBC and LDH are established markers of systemic inflammation and tissue damage in severe COVID-19 [3, 24, 25]. In contrast, in the older group (≥ 65 years), the CD147hi cMono frequency was significantly correlated with steroid treatment duration and showed a positive correlation trend with CXCL10 levels.The association with steroid treatment in older patients may reflect that higher CD147hi cMono levels are present in patients requiring more intensive immunosuppression. The trend towards correlation with CXCL10 suggests a potential role for CD147hi cMono in chemokine-mediated immune cell recruitment in older individuals, although this requires further study with larger sample sizes. While the correlation between CD147hi cMono and CXCL10 showed a trend in the older age group, the correlation with the overall severity score was not significant in this group, suggesting that in older individuals, other age-related factors might play a more dominant role in determining overall disease severity, although CD147hi cMono may still contribute to specific aspects of inflammation such as CXCL10 production. Previous studies have shown age-related changes in immune cell function [26], and immune cell responsiveness to S1 stimulation may be also modulated by age. Further research is needed to investigate the relationships among age, CD147 expression, and COVID-19 severity.

The identification of CD147hi cMono as key inflammatory cells has important clinical implications. The proportion of these cells, particularly in younger individuals, could serve as a biomarker for disease severity and treatment response monitoring. This is particularly relevant given that their frequency decreases with clinical improvement, as observed in our follow-up samples. Additionally, our findings provide a rationale for therapeutic strategies targeting CD147, supported by promising results from anti-CD147 antibody trials in severe COVID-19 [13, 14].

Recent evidence suggests that persistent S1 subunit might contribute to ongoing inflammation in some COVID-19 patients [27, 28]. Our identification of CD147hi cMono as key responders to S1 subunit might provide insights into these chronic inflammatory mechanisms, although further studies are needed to explore this possibility.

Several limitations of our study should be acknowledged. First, the precise molecular mechanisms by which CD147 expression on circulating monocytes enhances pro-inflammatory cytokine production, such as IL-1β, TNF-α, and IL-6, require further investigation. Second, our findings would benefit from validation in larger cohorts with a broader disease severity spectrum, including mild, moderate, severe, and critical COVID-19. Third, our analysis focused on circulating monocytes; the role of tissue-resident, CD147-expressing cells, particularly in the lungs, remains to be explored. Fourth, our analysis focused on the S1 subunit of SARS-CoV-2. Wick et al. reported that plasma SARS-CoV-2 N-antigen concentrations correlated with IL-10 levels and were associated with ICU admission and 28-day mechanical ventilation [29]. Future studies should also investigate the role of other SARS-CoV-2 antigens, such as N-antigen, in relation to CD147hi cMono and COVID-19 severity. Fifth, our stratified analysis revealed age-specific correlation patterns. However, the relatively small sample size in each age group, particularly the younger group (*n* = 11), and the unavailability of BMI data, warrant cautious interpretation of these age-stratified results and highlight the need for validation in larger, age-stratified cohorts that include BMI assessment. Further studies with larger cohorts and mechanistic analyses are needed to validate these findings and explore the therapeutic potential of targeting CD147hi cMono in severe COVID-19.

## Conclusion

We identified CD147hi classical monocytes as the primary source of S1-induced inflammatory cytokines and their association with COVID-19 severity. The proportion of CD147hi cMono increases during acute inflammation and decreases with clinical improvement, suggesting its utility as a biomarker for disease monitoring. Our age-stratified analysis revealed distinct correlation patterns in younger and older patients, highlighting the age-dependent clinical relevance of CD147hi cMono in COVID-19. These findings provide new insights into the cellular basis of COVID-19 pathogenesis and suggest that CD147 is a potential therapeutic target, particularly in the context of age-specific therapeutic strategies.

## Supplementary Information

Supplementary Material 1: Table S1. Demographic and clinical characteristics of study participants. Table S2 List of antibodies used for CyTOF cytokine panel in vitro assay. Table S3 List of antibodies used for CyTOF surface marker analysis of COVID-19 patient samples. Table S4 Spearman correlation coefficients between CD147hi cMono frequency and other variables, stratified by age group (< 65 years and ≧65 years). *p < 0.05, **p < 0.01, ***p < 0.001.

Supplementary Material 2: Supplementary Fig. 1. Additional cytokine analysis data and correlations. a. Serum levels of additional cytokines in patients with COVID-19 and HDs. Each value is indicated by a dot and the median by a bar. Pairwise comparisons were performed using the Kruskal–Wallis test. b. Correlation analysis between serum S1 subunit levels and clinical parameters. Heatmap shows Spearman's correlation coefficients, and asterisks indicate *p*-values (*p < 0.05, **p < 0.01, ***p < 0.001). c. Additional cytokine measurements in S1 subunit-stimulated PBMC culture supernatants. Significance was determined by the Kruskal–Wallis test, and bars indicate medians. Supplementary Fig. 2. Additional CyTOF analysis of S1 subunit stimulation experiments. a. FlowSOM clustering heatmap showing expression profiles and percentages of major immune subsets. b. Expression of activation markers on myeloid cells. Box plots show interquartile ranges, and significance was determined by the Kruskal–Wallis test. c. CD147 expression analysis across myeloid subsets. Box plots show interquartile ranges, and significance was determined by the Kruskal–Wallis test. d. FlowSOM clustering heatmap of myeloid cell re-clustering. e. Changes in monocyte subset proportions following S1 subunit stimulation. Significance was determined by the Kruskal–Wallis test. Supplementary Fig. 3. Additional CyTOF analysis of COVID-19 patient samples. a. UMAP visualization of major immune populations from patient PBMC analysis. b. FlowSOM clustering heatmap showing major immune subset profiles. c. Analysis of monocyte subset proportions. Significance was determined by the Kruskal–Wallis test. d. Correlation analysis between S1 subunit levels and clinical parameters. Graphs show Spearman's correlation coefficients and *p*-values.

## Data Availability

The datasets used and/or analyzed during the current study are available from the corresponding author upon reasonable request.

## Abbreviations

ARDS: Acute respiratory distress syndrome
CAS: Cell Acquisition Solution
COVID-19: Coronavirus disease 2019
CSB: Cell Staining Buffer
CXCL10: C-X-C motif chemokine ligand 10
CyTOF: Cytometry by time-of-flight
EMMPRIN: Extracellular matrix metalloproteinase inducer
FBS: Fetal bovine serum
HD: Healthy donor
HIV: Human immunodeficiency virus
HRP: Horseradish peroxidase
ICU: Intensive Care Unit
IL: Interleukin
LDH: Lactate dehydrogenase
MAPK: Mitogen-activated protein kinase
mDC: Myeloid dendritic cell
NF-κB: Nuclear factor-kappa B
NLRP3: NLR family pyrin domain containing 3
PBMC: Peripheral blood mononuclear cell
PI3K/Akt: Phosphatidylinositol-3 kinase/ protein kinase B
RPMI: Roswell Park Memorial Institute
RT: Room temperature
SARS-CoV-2: Severe acute respiratory syndrome coronavirus 2
TLRs: Toll-like receptors
TNF: Tumor necrosis factor
UMAP: Uniform Manifold Approximation and Projection
WBC: White blood cells
WHO: World Health Organization
