# Supplementary figures and images for: CD147-high classical monocytes: a cellular biomarker for COVID-19 disease severity and treatment response
Source: Inflamm Regen. 2025 Apr 7;45:8. doi: 10.1186/s41232-025-00371-8 (PMC11974131; doi:10.1186/s41232-025-00371-8)

Supplement figure 1

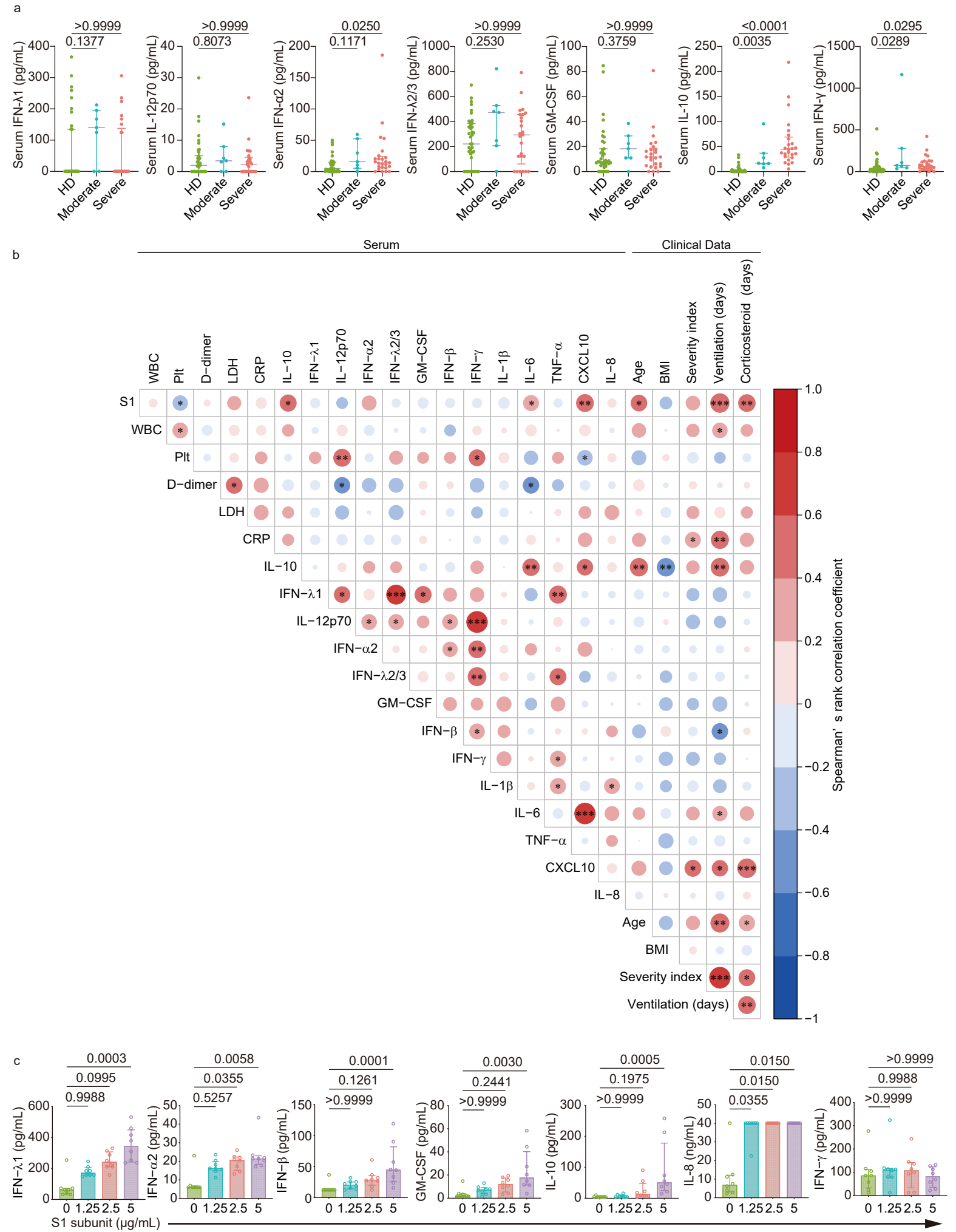

Supplement figure 2

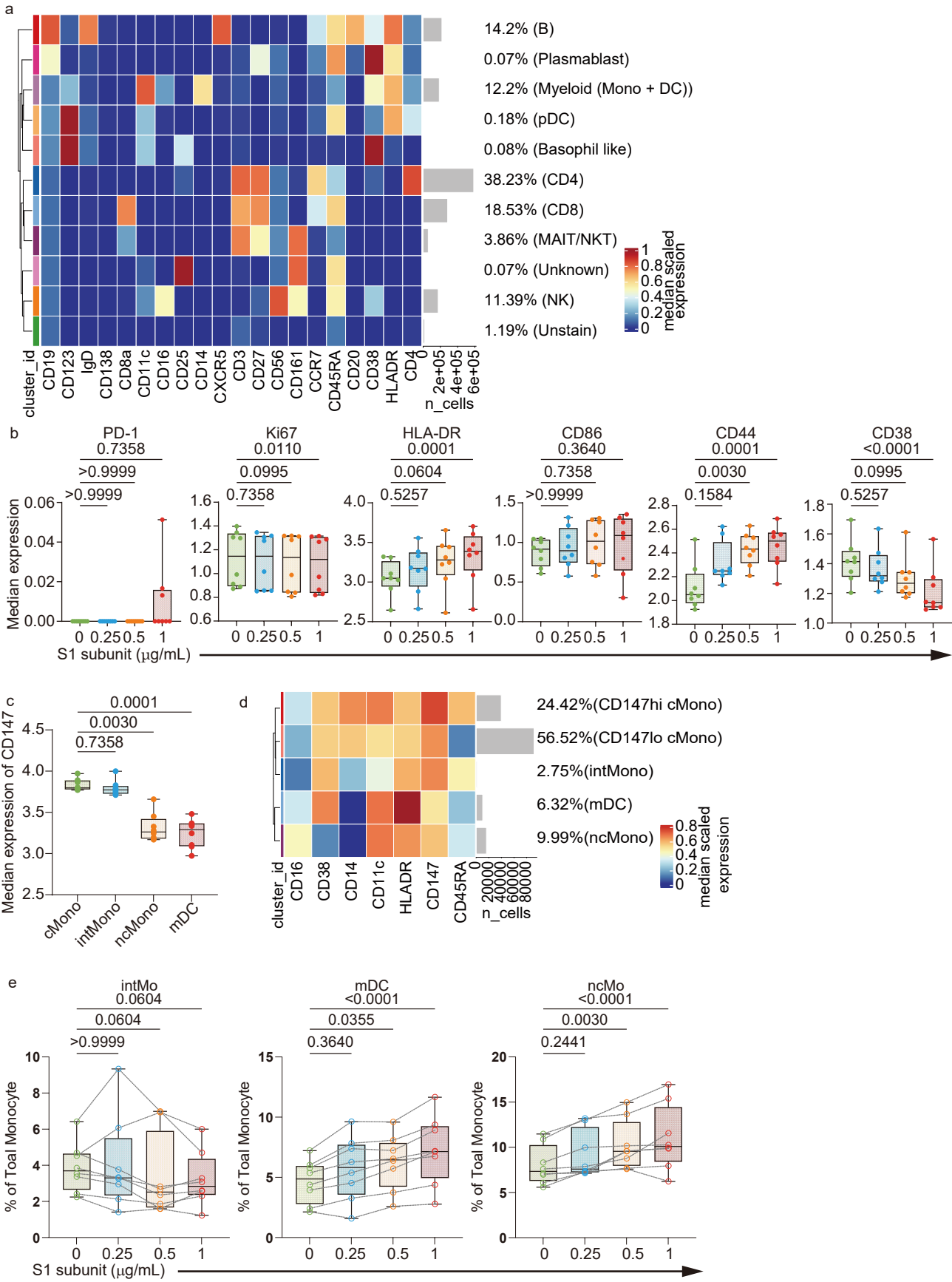

Supplement figure 3

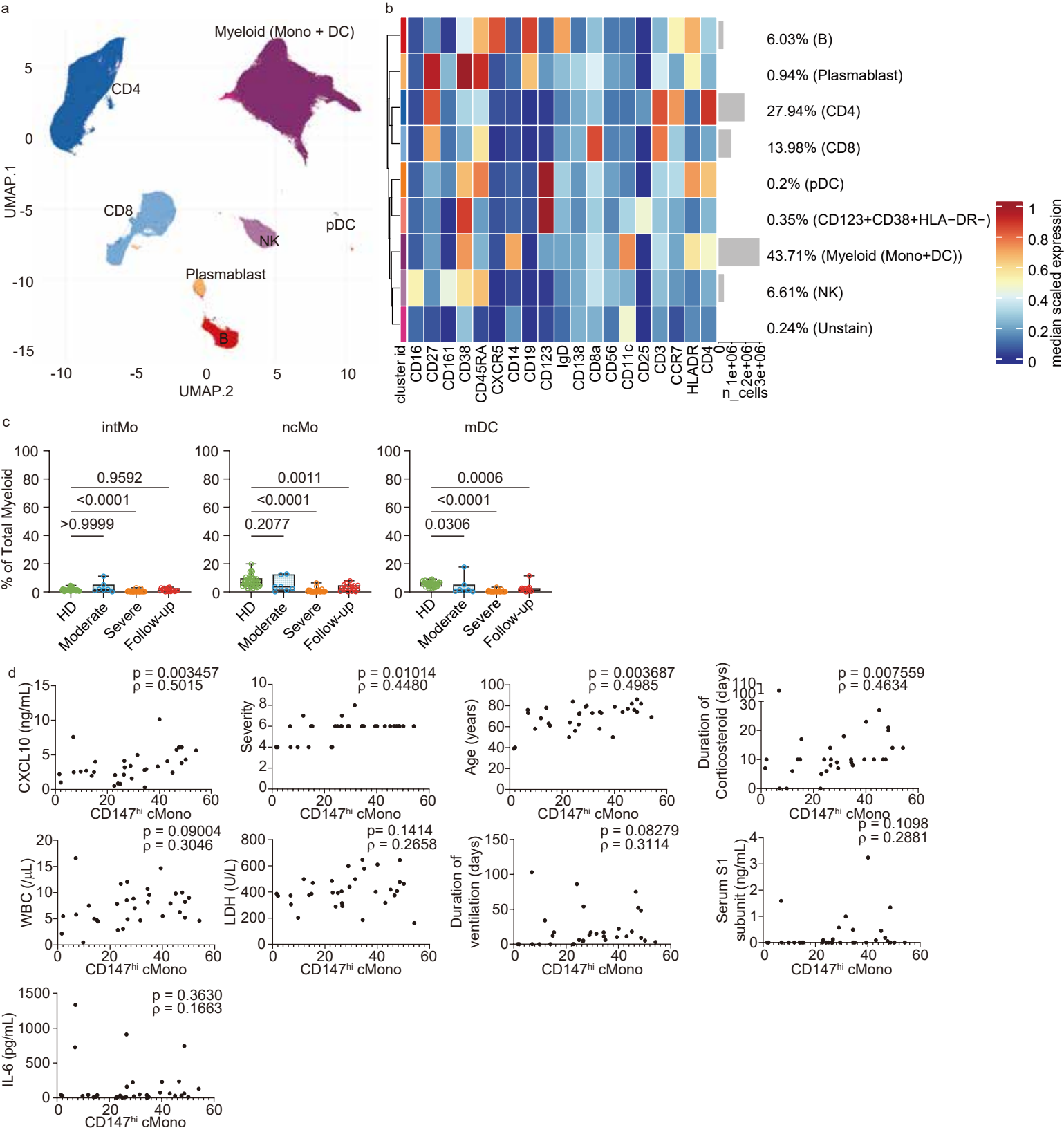

Supplement: Supplementary file 2 — Supplementary Material 2: Supplementary Fig. 1. Additional cytokine analysis data and correlations. a. Serum levels of additional cytokines in patients with COVID-19 and HDs. Each value is indicated by a dot and the median by a bar. Pairwise comparisons were performed using the Kruskal–Wallis test. b. Correlation analysis between serum S1 subunit levels and clinical parameters. Heatmap shows Spearman's correlation coefficients, and asterisks indicate p-values (*p < 0.05, **p < 0.01, ***p < 0.001). c. Additional cytokine measurements in S1 subunit-stimulated PBMC culture supernatants. Significance was determined by the Kruskal–Wallis test, and bars indicate medians. Supplementary Fig. 2. Additional CyTOF analysis of S1 subunit stimulation experiments. a. FlowSOM clustering heatmap showing expression profiles and percentages of major immune subsets. b. Expression of activation markers on myeloid cells. Box plots show interquartile ranges, and significance was determined by the Kruskal–Wallis test. c. CD147 expression analysis across myeloid subsets. Box plots show interquartile ranges, and significance was determined by the Kruskal–Wallis test. d. FlowSOM clustering heatmap of myeloid cell re-clustering. e. Changes in monocyte subset proportions following S1 subunit stimulation. Significance was determined by the Kruskal–Wallis test. Supplementary Fig. 3. Additional CyTOF analysis of COVID-19 patient samples. a. UMAP visualization of major immune populations from patient PBMC analysis. b. FlowSOM clustering heatmap showing major immune subset profiles. c. Analysis of monocyte subset proportions. Significance was determined by the Kruskal–Wallis test. d. Correlation analysis between S1 subunit levels and clinical parameters. Graphs show Spearman's correlation coefficients and p-values. [file 41232_2025_371_MOESM2_ESM.pdf]
